# Associations of Partnership Quality and Father-to-Child Attachment During the Peripartum Period. A Prospective-Longitudinal Study in Expectant Fathers

**DOI:** 10.3389/fpsyt.2021.572755

**Published:** 2021-04-20

**Authors:** Susanne Knappe, Johanna Petzoldt, Susan Garthus-Niegel, Julia Wittich, Hans-Christian Puls, Isabell Huttarsch, Julia Martini

**Affiliations:** ^1^Institute of Clinical Psychology and Psychotherapy, Technische Universität Dresden, Dresden, Germany; ^2^Department of Medicine, Faculty of Medicine, Medical School Hamburg, Hamburg, Germany; ^3^Department of Child Health and Development, Norwegian Institute of Public Health, Oslo, Norway; ^4^Institute and Policlinic of Occupational and Social Medicine, Faculty of Medicine, Technische Universität Dresden, Dresden, Germany; ^5^Department of Psychiatry and Psychotherapy, Faculty of Medicine, Carl Gustav Carus University Hospital, Technische Universität Dresden, Dresden, Germany

**Keywords:** paternal attachment, paternal anxiety or depression, partnership quality, peripartum, fatherhood, pregnancy, postpartum

## Abstract

**Background:** During the transition to parenthood, a complex network of relationships unfolds between father, mother and the child. Expectant parents begin bonding with their unborn child, with this antenatal process supposedly being predictive for later postnatal attachment and child mental health. At the same time, couples may experience a change in partnership quality. While the majority of previous studies focused on associations between psychopathology, partnership quality and attachment from the perspective of mothers, the changes in partnership quality and attachment from the perspective of fathers has gained far less attention.

**Methods:** Data were derived from the *M*aternal *A*nxiety and it's *R*elation to *I*nfants' Development (MARI) study. *N* = 109 expectant fathers were recruited during mid-pregnancy (22 to 26 week of gestation). Lifetime anxiety and depressive disorders (DSM-IV) were assessed with a standardized diagnostic interview (CIDI). Paternal partnership characteristics and father-to-child attachments were assessed using standardized questionnaires at the second trimester, 10 days after delivery and 4 months after delivery in *N* = 76 fathers. Analyses were based on bivariate, robust and multivariate regression analyses.

**Results:** Fathers did not report an overall decrease in partnership quality during the peripartum period. However, fathers with comorbid anxiety and depressive disorders reported lower partnership satisfaction at postpartum, as compared to unaffected fathers. Fathers with pure depressive disorders reported lower intensity of antenatal attachment. Paternal antenatal partnership quality was positively associated with antenatal father-to-child attachment. Furthermore, antenatal father-to-child attachment, as well as ante- and postnatal partnership quality in fathers, were positively related to postnatal father-to-child attachment.

**Conclusions:** Antenatal father-to-child-attachment and paternal partnership quality appear to be promising targets for the prevention of postnatal attachment problems in fathers. The associations between partnership quality and attachment to the child further support an interpersonal approach in perinatal research, treatment and intervention, and may also feed into awareness programs that encourage expectant fathers to actively engage in relationships as early as during pregnancy—both with the mother and the unborn child.

## Introduction

A range of studies has demonstrated that the transition into parenthood is associated with substantial changes such as an increased psychopathological vulnerability (1), changes in partnership quality (2–9), and the development of an emotional bond between parent and child (10). The majority of studies to date has focused on mothers and their child (11). Albeit the role of fathers has gained more importance in recent years (12–15), evidence from the perspective of expectant fathers on the concomitant developments during the peripartum period is limited. In contrast to mothers, (expectant) fathers are often harder to reach for perinatal research studies, entailing the risk of a selection bias. Nonetheless, recruitment of only complete families (i.e., mothers, fathers, and children participating jointly) may not reflect the reality of family lifestyles and relationships. Thus, it has to be kept in mind that the participation of fathers in research is limited by selection and recruitment barriers.

For expectant mothers, prevalence rates and outcomes for depressive and anxiety disorders during pregnancy have been widely reported to be higher, when compared to the general female population [e.g., (16–20)]. Similarly, expectant fathers also appear to have an increased risk of depression in the perinatal period (12, 20, 21). While the prevalence rate of depression in men in the general population ranges between 3 and 6% (22–24), a meta-analysis found that the rate of paternal depression between the first trimester and 1 year postpartum was 10.4% (14), which was also observed recently in a sample of expectant fathers (25). There is also evidence for higher rates of anxiety disorders during pregnancy in both mothers and fathers (26). Further, maternal and paternal anxiety and depressive disorders appear to be correlated, and may even accumulate in couples (14, 27, 28). Some studies have also found support for paternal-specific course patterns. For example, Leach et al. (29) did not find higher rates of paternal depressive and anxiety disorders in the peripartum period of up to 12 months after birth, but in the periods before and after. However, conclusions on the impact of paternal psychopathology during this period, and its complex interplay with psychosocial variables, are hampered by diverging study designs, assessment strategies, and the lack of prospective longitudinal studies.

Peripartum anxiety and depressive disorders are often associated with significant impairment and distress in mothers and fathers, which may be reflected in altered partnership quality (6, 14, 30–33). For instance, depressed spouses reported both lower initial satisfaction and a higher decline in satisfaction from the pre- to postpartum period (3). Additionally, couples with a depressed partner were characterized by lower satisfaction and more dysfunctional interaction patterns, including lower communication and problem-solving ability, lower social support, less self-disclosure and intimacy, higher negativity, and a stronger focus on bodily and psychological complaints (34–42). Cox et al. (3) similarly observed that marital satisfaction in couples was high antenatally, but decreased during transition to parenthood until the end of the child's first year.

During transition to parenthood, interactions between spouses become even more dynamic as the child and the (expectant) parents gradually attach themselves to each other in a triadic relationship (43–48). Within these relationships, a correlating psychopathology of mothers and fathers represents a further complication (14). An analysis in the MARI study on (expectant) mothers showed that women with comorbid panic disorder and major depression during the peripartum period reported lower partnership quality and impaired postpartum bonding to their child (49). However, attachment and bonding research has suffered from the inconsistent use of varying terms and concepts to describe these emotional relationships, focusing predominantly on the interaction between mother and child (50–52). Nonetheless, paternal attachment has been conceptualized as antenatal and postnatal attachment (53), and was found to be similarly important for child development up to adolescence and young adulthood as maternal attachment. For example, unpredictable relationships and insecure father-to-child attachment were linked to externalizing and internalizing mental disorders in adolescence [e.g., (54, 55)]. Secure paternal attachment was also found to compensate for impaired child-to-mother attachment (56, 57). Göbel et al. (58) recently reported that fathers with higher levels of avoidant attachment reported lower bonding intensity. Albeit these latter findings were based on cross-sectional analyses in small samples, they point to the dynamic associations between paternal well-being, partnership characteristics, and attachment to the child. Nevertheless, evidence on paternal attachment and its interplay with paternal mental health and partnership characteristics from prospective studies remains limited. Studies on perinatal mental health will most likely benefit from prospective insights into the nature, course, and interplay of these factors from the perspective of fathers. Hence, we aim to (1) depict changes in partnership quality in fathers across the peripartum period and (2) determine cross-sectional and prospective associations between partnership quality during this period with antenatal and postnatal father-to-child attachment. For both aims, the role of paternal anxiety and depressive disorders will be considered. We expect (H1) postnatal partnership quality to be lower than antenatal partnership quality, (H2) positive associations between antenatal and postnatal father-to child attachment, and (H3) positive associations between partnership quality and father-to-child-attachment. The role of paternal anxiety and depressive disorders, alone and in combination with maternal anxiety and depressive disorders, will be explored based on mixed findings from previous research.

## Materials and Methods

The *M*aternal *A*nxiety in *R*elation to *I*nfant Development (MARI; 01/2009–09/2012). Study includes a prospective-longitudinal study of (expectant) mothers (expectant), fathers, and their children, based on a regional epidemiological sampling of pregnant women and their partners.

A total of 533 pregnant women were approached by the study team in 22 gynecological outpatient settings in the area of Dresden (Germany) and screened for inclusion and exclusion criteria. *N* = 306 pregnant women were eligible during early pregnancy from January 2009 until June 2010. The MARI Study was carried out in accordance with the American Psychological Association (APA) ethical standards and was approved by the Ethics Committee of the Medical Faculty of the Technische Universität Dresden (No: EK 94042007). For further information on methods and design of the study, see Martini et al. (18, 19).

### The MARI Father Study

The MARI father study was designed to investigate the physical and mental health of expectant fathers during the transition to fatherhood, with special focus on paternal psychopathology and psychosocial correlates of child development. Fathers were assessed via face-to-face interviews and mailed questionnaires at three assessment points, namely at week 22 to 24 of gestation (F-T1), at 10 days after delivery (F-T2), and 4 months after delivery (F-T3). At F-T1 and F-T3, paternal psychopathology was comprehensively assessed using the standardized Composite International Diagnostic Interview (CIDI) (59, 60). Psychosocial variables, such as partnership quality or attachment, were assessed using questionnaires and response booklets embedded in the CIDI. At F-T2, birth related information (e.g., the fathers' experience of childbirth and postnatal mood) was assessed using questionnaires.

### Recruitment of Expectant Fathers and Participant Flow

**Figure 1:**
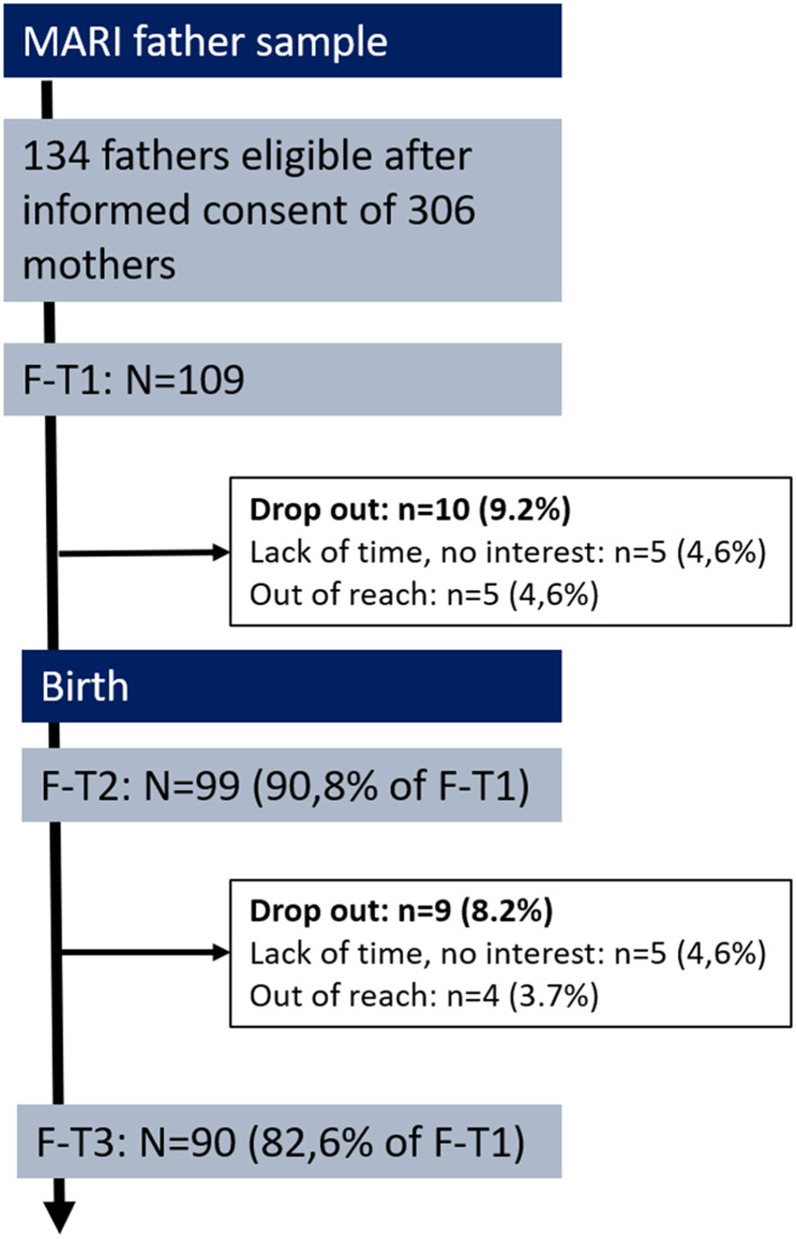
Flow chart and retension rates of the MARI father study. F-T1 week 22 to 24 of gestation, F-T2 at 10 days postpartum, F-T3 at 4 months postpartum.

At week 22 to 24 of gestation (expectant), mothers were asked to contact their partner for participation in the MARI study. *N* = 134 (out of 306, 43.8%) gave informed consent for the study team to contact the (expectant) father by phone. Fathers were then informed about the study aims, protocol, data handling, and voluntariness. Inclusion criteria for fathers were sufficient mastery of the German language, willingness, and time for participation. Of the *N* = 134 fathers approached by the study team, *n* = 109 both met the inclusion criteria and provided informed consent to participate (Figure 1). Fathers received 10 € as incentive for participation and a standard gift also valued approximately 10 €. Due to the lack of a genetic paternity test, mothers were asked at T2 about the biological father of the unborn child. For all 109 fathers, mothers confirmed biological paternity.

Within the total study population of the MARI study (*n* = 306), we found substantial differences in sociodemographic, neonatal, and psychosocial characteristics between mothers participating with (*n* = 109/306) vs. without a partner (*n* = 197/306) (Supplementary Table 1). In the sample of parents participating jointly, mothers were significantly younger and both parents were significantly better educated. Infants had a significantly higher birth weight and were more often the first child of their parents. Also, both parents reported a significantly higher social support after delivery and a higher partnership quality before and after delivery.

### Variables Assessed

#### Paternal Anxiety and Depressive Disorders

Diagnostic assessment of paternal anxiety and depressive disorders was based on the Computer-Assisted Personal Interview (CAPI) version of the Composite International Diagnostic Interview [CIDI; (60)]. The CIDI is a modified version of the World Health Organization CIDI [WHO-CIDI: (61)] and allows for a fully standardized assessment of symptoms, syndromes, and diagnoses of DSM-IV-TR/ ICD-10 mental disorders with established reliability, procedural validity, and excellent psychometric properties in the assessment of anxiety and depressive disorders (62, 63). At F-T1, paternal psychopathology prior to conception was assessed with the lifetime version of the CIDI, including information regarding onset, recency, severity, comorbidity, and impairment of anxiety and depressive disorders. At follow-up assessments, the interval version of the CIDI was used to assess the course of symptoms and syndromes throughout pregnancy and after delivery. At each assessment point, participants completed several additional questionnaires to assess the course of psychopathological symptoms and further relevant constructs [see (18)]. Interviews were conducted by psychologists who had received 1 week of intensive training, including the CIDI standard training (60). The interviewers were monitored closely throughout the field period by experienced supervisors (clinical psychologists). Based on lifetime diagnostic information at F-T1, participants were allocated to one of the following initial diagnostic groups, equivalent to the MARI main study [cf. (18, 30)]: no anxiety nor depressive disorder prior to pregnancy; pure depressive disorder(s) prior to pregnancy or pure anxiety disorder(s) prior to pregnancy; comorbid anxiety and depressive disorders prior to pregnancy. To further depict the psychopathological load in couples, information about maternal psychopathology was considered as well [cf. (18)] resulting in three more diagnostic groups, namely no partner affected, one partner affected, or both partners affected by anxiety or depressive disorders.

### Partnership Quality

Partnership characteristics were assessed among all expectant fathers who indicated to currently having a partner at F-T1 and F-T3 using the Partnership Questionnaire [PFB; (64)]. The PFB contains 30 items (labeled never/very seldom, seldom, often, and very often) assessing the dimensions communication (10 items), quarreling (10 items), and tenderness (10 items). The individual subscales can be accumulated to a total PFB score (overall partnership quality). An additional single item (labeled very happy, happy, somewhat happy, somewhat unhappy, unhappy, and very unhappy) assesses current partnership satisfaction (“At this moment, how happy do you think your relationship is?”). The three-dimensional factor structure of the PFB has been consistently confirmed. Internal consistency (Cronbach's α = 0.88–0.95) and retest-reliability (range 0.68–0.85; *N* = 534), as well as discriminant and predictive validity of the PFB have been shown to be high (64–69).

### Father-to-Child Attachment

Father-to-child attachment was assessed in fathers using the German version of the 16-item Paternal Antenatal Attachment Scale [PAAS; (70)] at F-T1 and the 19-item Postnatal Paternal-Infant Attachment Scale [PPAS; (71)] at F-T3. Responses were collected on an item-specific five-point Likert Scale, with one representing the absence of feelings and five representing very strong feelings toward the child. Translation and back-translation of the original scales were part of the MARI study and based on a German adaption of the original PAAS and PPAS (72, 73).

The PAAS depicts the overall antenatal attachment of the father to his unborn child on a sum score and can be organized into two subscales, representing its underlying dimensions quality and quantity (intensity). Quality of attachment (six items) refers to antenatal paternal experiences of bonding, tenderness, and joy in the anticipated interaction with the child, as well as perceived stress when imagining the loss of the unborn. The subscale quantity of preoccupation (eight items) refers to the extent and intensity of the father's antenatal preoccupation with the unborn child (e.g., “Over the past 2 weeks I have found myself talking to my baby”). First evidence regarding the factorial, construct, and discriminant validity of the original PAAS (70) exists. Internal reliability of the PAAS sum score and subscales were high at Cronbach's α > 0.80 (45, 53). Recently, two factors equivalent to the original quality and intensity dimensions were identified for a German 13-item version of the PAAS; there, scale reliability for the extracted factors was satisfactory to good, with Cronbach's α ranging from 0.71 to 0.82 (74).

The PPAS refers to the postnatal attachment of the father to his child (here at 4 months postpartum), represented by a sum score comprising the three subscales “patience and tolerance,” “pleasure in interaction,” and “affection and pride.” According to the original PPAS (71), the scales represent the following underlying dimensions of the father-to-child attachment construct: absence of irritability and other negative affects (e.g., boredom) toward the child, as well as a lack of resentment about the personal impact of fatherhood (subscale “patience and tolerance”); experiences during actual interactions with the child, such as feelings of pleasure, satisfaction, and competence during “hands-on” interactions (e.g., the desire to prolong such an involvement) (subscale “pleasure in interaction”); stable and enduring ‘background’ feelings and cognitions toward the child (e.g., a sense of belonging to the child) (subscale “affection and pride”). Internal consistencies of the original PPAS and its subscales are sufficient to good (45, 71).

### Statistical Analyses

The software package Stata 14.1 (75) was used for all analyses. Linear regressions were used to test associations (standardized beta coefficients, β) between lifetime paternal psychopathology at F-T1 (Four initial diagnostic groups: no anxiety or depressive disorder, pure anxiety disorder, pure depressive disorder, comorbid anxiety and depressive disorder; three groups on psychopathological load of the couple: no partner affected, one partner affected, both partners affected) as predictor and partnership quality (overall partnership quality, quarreling, tenderness, communication, and satisfaction) at F-T1, F-T3 and changes (i.e., difference) from F-T1 to F-T3 as outcomes. Since partnership quality has been shown to be lower in older individuals (67), all analyses were adjusted for age at baseline. Robust regressions were used in the event of violations of assumptions for linear regressions. Linear regressions were further used to assess associations between lifetime paternal psychopathology at F-T1 with antenatal and postnatal father-to-child attachment. Pairwise Pearson Correlations were applied to test for correlations between partnership characteristics and antenatal and postnatal father-to-child attachment. Univariate and multivariate linear regressions were used to test for the predictive value of antennal partnership quality and father-to-child attachment for postnatal status. *T*-tests for independent groups were applied to compare partnership characteristics and father-to-child attachment between first-time fathers and experienced fathers. Statistical analyses refer to *n* = 76 (expectant) fathers who indicated they currently live in a relationship with the mother of the child and subsequently provided information on their partnership quality at F-T1 or F-T3.

## Results

### Sample Characteristics

Sample characteristics of fathers are listed in Table 1. Mean age of fathers was 31.1 years (SD=5.79, Range 20–51 years) at F-T1. Mean household income (net wage) ranged between 1,500 and 2,500 € which is representative for mid-income families in this region of Germany. More than half of the fathers were unmarried. Only one father was not living together with the mother of his child at study entry, while the majority of fathers (93.1%) were in a relationship with their child's mother. About 2/3 of the fathers were first-time-fathers; 65.2% of the fathers reported that the pregnancy was planned and 71.6% indicated that the pregnancy was wanted.

**Table 1:** Sample characteristics of the MARI-father study at F-T1.

		**Total father sample** ** (*****N*** **=** **109)**		**Fathers with information on partnership and attachment** ** (*****N*** **=** **76)**		**Group** ** difference**	
		***n***	**%**	***n***	**%**	**χ^2^**	***p*-value**
Age in years	20–29	53	48.6	33	43.4	*T* = 1.6905, df = 107, *p* = 0.0938	
	30–39	45	41.3	35	46.1		
	40–49	10	0.9	7	9.2		
	50–51	1	0.9	1	1.3		
Marital status	Married	42	38.6	34	44.7	5.6	0.087
	Separated or divorced	4	3.6	3	4.0		
	Never married	63	57.8	39	51.3		
Living together with partner at F-T1	Yes	102	93.6	76	100.0	2.8	0.262
	No	1	0.9	0	0.0		
	Missing	6	5.5				
Number of prior deliveries in female partner	0	71	65.1	48	63.2	1.1	0.664
	1	29	26.6	21	27.6		
	2 or more	9	8.3	7	9.2		
Education^a^	No degree	2	1.8	1	1.3	1.3	0.777
	9th grade	5	4.6	3	4.0		
	10th grade	26	23.9	20	26.3		
	High school	34	31.2	23	30.3		
	University	42	38.5	29	38.2		
Current occupation^a^, ^b^	Unemployed	6	5.5	3	4.0	1.2	0.364
	Employed	76	69.7	58		2.3	0.173
	Student	22	20.2	12	15.8	3.0	0.118
	Other	8	7.4	5	6.6	0.2	0.696
Monthly household income in € after taxes^a^	Less than 500	9	8.3	4	5.3	5.8	0.406
	500 to 1,500	46	42.2	30	39.5		
	1,500 to 2,500	30	27.5	22	29.0		
	2,500 to 3,500	16	14.7	13	17.1		
	3,500 to 4,500	6	5.5	5	6.6		
	More than 4,500	2	1.8	2	2.6		
Planned pregnancy	For quite some time	39	35.8	31	40.8	6.1	0.116
	Currently planned	32	29.4	26	34.2		
	Planned for later	18	16.5	12	15.8		
	Not planned at all	14	12.8	7	9.2		
	Missing	6	5.5	0	0.0		
Wanted pregnancy	Very much	78	71.6	58	76.3	1.3	0.741
	Much	21	19.3	16	21.1		
	Rather not	3	2.7	1	1.3		
	Not at all	1	0.9	1	1.3		
	Missing	6	5.5	0	0.0		
Biological paternity^a^	Yes	109	100.0	76.0	100.0	n.a.	
	No	0	0.0	0.0	0.0		
Paternal psychopathology	No anxiety or depressive disorder	62	56.8	45	59.2	1.3	0.741
	Anxiety disorder only	32	29.4	20	26.3		
	Depressive disorder only	5	4.6	4	5.3		
	Comorbid anxiety and depressive disorder	10	9.2	7	9.2		
Psychopathological load	No anxiety or depressive disorder in either parent	25	22.9	19	20.0	1.4	0.160
	One parent affected	54	49.5	37	48.7		
	Both parents affected	30	27.5	20	26.3		

a *Indirect information from expectant mothers*.

b *Multiple choice*.

*N, number; %, percentages. n.a., not applicable; X^2^, Chi Square Test; p, significance level at 0.05*.

At F-T1, 56.8% (*n* = 62/109) of the fathers did not report any lifetime anxiety or depressive disorder. Rates for any lifetime anxiety disorder was 29.4% (*n* = 32/109), while the rates for any depressive disorder was 4.6% (*n* = 5/109); 9.2% (*n* = 10/109) reported comorbid anxiety and depressive disorder.

Fathers who could be retained in the study throughout the peripartum period (*N* = 76) did not differ from fathers in the total sample with regard to partnership quality and father-to-child attachment (Table 1). Further, there was no selective drop-out of fathers with any anxiety or depressive disorder after F-T1 (Pearson Chi2 = 1.8499; Fisher's Exact = 0.928) or F-T2 (Pearson Chi2 = 2.1136; Fisher's Exact = 0.724). No indications were found for assortative mating in terms of higher participation rate in fathers when mothers reported a lifetime anxiety or depressive disorder (Pearson Chi2 = 0.4532; Fisher's Exact = 0.938).

With regard to the initial diagnostic groups, *n* = 45/76 reported no anxiety or depressive disorder prior to pregnancy, *n* = 4/76 reported pure depressive disorder(s) prior to pregnancy, *n* = 20/76 reported pure anxiety disorder(s) prior to pregnancy, and *n* = 7/76 reported comorbid anxiety and depressive disorders prior to pregnancy. For psychopathological load in couples, no lifetime anxiety or depressive disorder prior to pregnancy was reported for either mother or father in *n* = 19/76 cases. In *n* = 37/76 cases, one parent was affected (i.e., lifetime anxiety or depressive disorder prior to pregnancy in either mother or father). Lifetime anxiety or depressive disorder prior to pregnancy in both mother and father was observed in *n* = 20/76 cases.

### Partnership Quality During the Transition to Fatherhood (H1)

Means and standard deviations for partnership quality at F-T1, F-T3, and across time are presented in (Supplementary Table 2). Fathers did not report any decrease in partnership quality across the peripartum period, albeit there was a tendency for fathers to report less tenderness (*p* = 0.063). Fathers also reported less satisfaction at F-T3 than at F-T1 (*t* = 2.87, df = 73, *p* = 0.005).

First-time fathers (*n* = 48/76) and experienced fathers did not differ on PFB sores at F-T1, F-T3, or throughout the peripartum period. However, fathers who scored lower on partnership quality at F-T1 had a higher chance to drop-out during the MARI father study on F-T2 or F-T3. This was shown through lower scores on the PFB subscale communication (*N* = 76; *t* = 2.10, *p* = 0.038) and in terms of lower satisfaction with partnership (*N* = 76, *t* = 2.39, *p* = 0.019).

Taking lifetime paternal psychopathology into account, fathers with comorbid anxiety and depressive disorders prior to pregnancy reported lower partnership satisfaction at postpartum (Beta = −1.65, 95% CI: −2.48 to *n* = 0.82, *p* ≤ 0.050), as compared to unaffected fathers (Table 2). With regard to changes in partnership quality across the peripartum period, i.e., from F-T1 to F-T3, fathers with pure depression reported increasing levels of quarreling (Beta = 0.40, 95% CI: 0.32 to 1.93, *p* = 0.007). For changes in overall partnership quality, other PFB subscales and satisfaction, no associations with paternal psychopathology were observed (Table 2).

**Table 2:** Prospective associations of paternal psychopathology with paternal partnership quality and changes across peripartum.

		**F-T1**		**F-T3**		**Prospective associations with F-T3**					**Changes in F-T3**		**Prospective associations with changes across peripartum (F-T3–F-T1)**				
		**M**	**SD**	**M**	**SD**	**b**	***t***	***p***	**95% CI**		**M**	**SD**	**b**	***t***	***p***	**95% CI**	
PFB sum score^a^	No AD	68.77	11.40	68.55	12.46						−0.21	7.90					
	Pure A	69.24	10.89	66.96	12.19	−0.33	−1.17	0.240	−0.91	0.24	−2.28	7.89	0.00	0.03	0.978	−0.47	0.48
	Pure D	66.00	11.03	63.19	11.84	−0.18	−0.32	0.750	−1.29	0.93	−2.81	8.45	−0.60	−1.30	0.196	−1.53	0.32
	Comorbid AD	66.77	8.32	64.31	12.55	−0.15	−0.33	0.740	−1.02	0.74	2.46	7.53	−0.10	−0.27	0.785	−0.84	0.64
Communication^a^	No AD	23.30	3.77	23.86	4.52						0.56	3.57					
	Pure A	22.62	4.33	22.24	4.45	−0.15	−0.53	0.980	−0.72	0.42	−0.38	3.22	0.03	0.11	0.915	−0.45	0.50
	Pure D	21.50	4.03	22.33	4.40	−0.69	−1.25	0.216	−1.8	0.41	0.83	2.66	−0.64	−1.41	0.163	−1.56	0.27
	Comorbid AD	23.00	3.34	20.69	0.47	0.62	0.14	0.889	−0.82	0.94	2.31	3.59		−0.36	0.719	−0.09	0.60
Quarreling^a^	No AD	6.03	4.41	6.47	4.84						0.44	3.00					
	Pure A	4.48	3.93	5.29	4.04	−0.34	1.30	0.198	−0.18	0.87	0.81	2.27	0.20	0.95	0.344	−0.22	0.61
	Pure D	3.83	2.52	6.00	5.67	0.27	0.53	0.597	−0.75	1.29	2.17	5.41	**0.40**	**2.80**	**0.007**	**0.32**	**1.93**
	Comorbid AD	5.54	3.57	4.85	3.95	0.58	1.42	0.161	−0.23	1.39	−0.69	2.53	0.32	−0.08	0.940	−0.67	0.61
Tenderness^a^	No AD	21.50	5.22	21.17	5.91						−0.33	4.03					
	Pure A	20.95	4.70	20.01	5.71	−0.34	−1.19	0.239	−0.92	0.23	−1.09	3.80	0.13	0.55	0.583	−0.35	0.62
	Pure D	18.33	6.84	16.85	5.98	−0.25	−0.44	0.661	−1.36	0.87	1.48	3.90	−0.07	−0.15	0.884	−1.00	0.87
	Comorbid AD	19.31	4.64	18.46	6.17	0.25	0.57	0.569	−0.63	1.14	−0.85	3.11	0.21	0.57	0.573	−0.53	0.96
Satisfaction	No AD	4.52	0.74	4.24	0.87						−0.21	0.96					
	Pure A	4.57	0.75	4.24	0.94	−0.18	−0.65	0.517	−0.73	0.37	0.33	0.91	0.70	0.57	0.572	−0.34	0.61
	Pure D	4.42	0.51	4.18	0.60	0.72	1.39	0.170	−0.32	1.77	0.18	0.60	0.14	0.12	0.908	−0.86	0.96
	Comorbid AD	4.62	0.51	4.15	0.80	−1.65	−3.97	**<0.050**	−2.48	−0.82	−0.46	0.78		−1.02	0.310	−1.16	0.38

a *Results based on robust regression*.

*PFB partnership quality questionnaire, pure A anxiety disorder only, pure D depressive disorder only, comorbid AD comorbid anxiety and depressive disorder*.

*b, unstandardised regression coefficient; M, mean, SD, standard deviation; t, T-Test; CI, confidence interval; p, significance level at 0.05. Bold values indicate statistical significance at p < 0.05. F–T1 week 22 to 24 of gestation; F–T2 at 10 days postpartum, F–T3 at 4 months postpartum*.

Psychopathology in couples (i.e., no parent, one parent, both parents affected) was not associated with overall partnership quality and satisfaction at F-T3, and did not predict any changes in partnership quality from F-T1 to F-T3 (Supplementary Table 3).

### Father-to-Child Attachment During the Transition to Fatherhood (H2)

Mean scores and standard deviations on antenatal and postnatal father-to-child attachment are presented in (Supplementary Table 4). As expected, antenatal and postnatal father-to-child attachment were strongly interrelated (*r* = 0.64 for sum scores, *p* < 0.001).

No differences emerged for antenatal and postnatal attachment between first-time fathers and experienced fathers (details available upon request).

Taking lifetime paternal psychopathology into account, fathers with pure depressive disorders reported lower intensity of antenatal attachment (Beta = −2.77; 95% CI: −4.91 to −0.62, *p* ≤ 0.050), as compared to unaffected fathers (Table 3).

**Table 3:** Prospective associations of paternal psychopathology with antenatal and postnatal father-to-child attachment.

		***N***	**Mean**	**SD**	**B**	**t**	***p***	**95% CI**	
**Antenatal attachment (PAAS)**									
PAAS sum score	No AD	30	62.90	5.54					
	Pure A	21	62.71	6.32	−0.18	−0.11	0.911	−3.37	3.01
	Pure D	12	60.00	5.98	**4.17**	–**2.09**	**0.041**	–**8.15**	–**0.18**
	Comorbid AD	13	62.62	5.36	1.38	−0.72	0.477	−5.22	2.46
PAAS quality of attachment	No AD	30	35.70	2.87					
	Pure A	21	35.14	3.15	−0.55	−0.72	0.473	−2.08	0.98
	Pure D	12	35.25	2.86	1.20	−1.25	0.215	−3.10	0.71
	Comorbid AD	13	35.15	2.03	1.19	−1.29	0.201	−3.03	0.65
PAAS intensity of attachment	No AD	30	18.77	2.88					
	Pure A	21	18.86	3.17	0.09	0.11	0.915	−1.63	1.81
	Pure D	12	16.33	2.71	–**2.77**	–**2.57**	**0.012**	–**4.91**	–**0.62**
	Comorbid AD	13	18.46	3.43	0.59	−0.57	0.570	−2.66	1.47
**Postnatal attachment (PPAS)**									
PPAS sum score	No AD	30	75.97	9.10					
	Pure A	21	74.24	1.03	−1.73	−0.68	0.496	−6.62	3.31
	Pure D	12	76.13	7.08	−0.43	−0.14	0.892	−6.71	5.85
	Comorbid AD	13	77.05	6.82	0.58	0.19	0.850	−5.48	6.63
PPAS patience and tolerance	No AD	30	31.53	4.47					
	Pure A	21	32.48	4.44	0.95	0.80	0.424	−1.41	3.31
	Pure D	12	32.45	3.59	0.39	0.27	0.791	−2.55	3.33
	Comorbid AD	13	32.86	3.42	0.87	0.62	0.540	−1.96	3.71
PPAS pleasure in interaction	No AD	30	25.55	4.48					
	Pure A	21	23.35	4.58	−2.20	−1.81	0.074	−4.63	0.22
	Pure D	12	24.49	3.84	−1.07	−0.70	0.483	−4.10	1.96
	Comorbid AD	13	25.24	3.37	−0.32	−0.22	0.826	−3.24	2.60
PPAS affection an pride	No AD	30	18.89	1.61					
	Pure A	21	18.41	2.32	−0.47	−0.97	0.333	−1.44	0.49
	Pure D	12	19.18	0.72	0.25	0.41	0.682	−0.96	1.45
	Comorbid AD	13	18.95	1.21	0.03	0.04	0.965	−1.14	1.19

*PAAS, antenatal father-to-child-attachment; PPAS, postpartal father-to-child-attachment; pure A anxiety disorder only; pure D depressive disorder only; comorbid AD comorbid anxiety and depressive disorder. b, unstandardised regression coefficient; M, mean; SD, standard deviation; t, T-Test; CI, confidence interval; p, significance level at 0.05. Bold prints indicate statistical significance at p < 0.05*.

Considering psychopathological load, antenatal quality of attachment (Beta = −1.86, 95% CI: −3.60 to −0.11, *p* = 0.037) and postnatal pleasure in interaction (Beta = −3.35, 95% CI: −6.00 to −0.70, *p* = 0.014) was were lower when both parents were affected, as compared to no parent being affected (Supplementary Table 5).

### Associations Between Partnership Quality and Father-to-Child Attachment (H3)

Both antenatal and postnatal father-to-child attachments were strongly related to partnership quality at F-T1 and F-T3. However, no correlations were observed between antenatal or postnatal attachment with changes in partnership quality across the peripartum period (Table 4).

**Table 4:** Intercorrelations between partnership quality and father-to-child-attachment.

	**PFB F-T1**		**PFB F-T3**		**PFB change from F-T1 to F-T3**		**PAAS F-T1**		**PPAS F-T3**	
	***r***	***p***	***r***	***P***	***r***	***P***	***r***	***p***	***r***	***p***
PFB F-T1	1.00									
PFB F-T3	**0.77**	**<0.001**	1.00							
PFB change	0.15	0.210	–**0.52**	**<0.001**	1.00					
PAAS F-T1	**0.46**	**<0.001**	**0.33**	**0.003**	0.10	0.400	1.00			
PPAS F-T3	**0.38**	**<0.001**	**0.27**	**0.018**	0.09	0.458	**0.64**	**<0.001**	1.00	

*r pairwise Pearson correlation, p < 0.05. Bold prints indicate statistical significance at p < 0.05*.

*PFB, partnership quality; PAAS, antenatal father-to-child-attachment; PPAS, postnatal father-to-child attachment; F–T1 week 22 to 24 of gestation; F–T3 at 4 months postpartum*.

Based on linear regressions, overall partnership quality at F-T1 was positively associated with overall antenatal father-to-child attachment, as well as with antenatal attachment quality and antenatal intensity of attachment (PAAS; Beta range between 0.27 and 0.44, *p* < 0.05), indicating higher levels of partnership quality to be associated with more favorable attachment.

In univariate regression models (Table 5), postnatal father-to-child attachment (PPAS) was predicted by antenatal father-to-child attachment, antenatal partnership quality at F-T1, and postnatal partnership-quality at F-T3. In a multivariate model, antenatal attachment (Beta=0.6, *T* = 5.98, *p* < 0.01) but not partnership quality (neither F-T1 nor F-T3) predicted postnatal attachment in fathers.

**Table 5:** Univariate associations of antenatal attachment and partnership quality with postnatal father-to-child attachment.

	**Postnatal father-to-child attachment (PPAS)**					
	**b**	**SE**	**Beta**	**t**	***p***	***R* ^**2**^**
PAAS F-T1 sum score	**0.97**	**0.13**	**0.64**	**7.21**	**<0.001**	**0.41**
PFB F-T1	**0.19**	**0.08**	**0.27**	**2.42**	**0.018**	**0.07**
PFB F-T3	**0.31**	**0.09**	**0.38**	**3.49**	**0.001**	**0.14**

*Univariate linear regressions; b, unstandardised regression coeffizient; SE, standard error; BETA, standardized regression coefficient; t, T-value; R^2^ variance explained; p, significance level at p < 0.05, bold prints indicate statistical significance at p < 0.05*.

*PFB partnership quality; PAAS antenatal father-to-child-attachment; PPAQ postnatal father-to-child attachment*.

Changes in partnership quality across the peripartum period was found to be unrelated to postnatal father-to-child attachment (Beta=0.09, *T* = 0.75, *p* = 0.458).

## Discussion

This prospective study placed special emphasis on the perspectives of fathers in the emerging relationships during the peripartum period. Specifically, we investigated associations between paternal antenatal and postnatal partnership quality, and father-to-child attachment. A range of associations between partnership quality and attachment, both cross-sectionally and prospectively were observed, providing insights into perinatal mental health in fathers.

In this sample of relatively healthy, supportive, and resilient families, we found these associations to be less striking than expected, particularly when compared to available data on associations in mothers of the full study sample.

For fathers, a reduction of partnership quality and satisfaction was observed to occur between antenatal and postnatal periods. However, these differences across time were not found to be statistically firm. This finding is in contrast to the results from the MARI main sample of mothers, where women with comorbid anxiety and depressive disorders reported less tenderness during pregnancy, less postpartum tenderness, less satisfaction, and less overall partnership quality, as well as a decrease in communication from pre- to post-partum periods (30). Since fathers reported generally lower partnership quality than mothers, small changes in partnership quality in fathers may have been more difficult to detect. Another explanation might be that mothers evaluate their partnership more positively, and communicate more openly about tenderness than their male partners (67). Still, levels of paternal partnership quality in this study were higher than in other samples (64, 67), which is likely a direct result of the selective sample of highly engaged, supportive fathers. In fact, fathers in our study were older than those from the reference data, more interested in study participation, and possibly more involved in the support of their offspring and partner. In addition, fathers who presented lower scores in the PFB communication and partnership satisfaction subscales were more likely to drop out at subsequent assessments.

Our study adds to previous research (45, 58), that father-to-infant attachment develops similarly as mother-to-infant attachment during the transition to parenthood. Furthermore, this is the first study that shows longitudinal associations between antenatal and postnatal attachments.

In accordance with the mothers of the full study sample, pure anxiety or depressive disorders in expectant fathers were unrelated to postpartum partnership quality. As only 5 out of 109 fathers (4 out of 76) were diagnosed with pure depression, this group was most likely too small to confirm previous findings on the relationships between paternal depressive disorders and attenuated partnership quality (35, 36, 38–42). Still, depressive fathers reported an increase of quarreling across the peripartum period which was also observed in the MARI mother sample (30) and other studies (35, 36, 38–42). These findings may be explained by less frequent and less in-depth conversations, higher levels of negativity and hostility, and limited problem solving skills in couples with a depressive partner (76, 77). No changes were found for other partnership characteristics such as communication, suggesting a possible decrease in partnership quality despite stable communication quantity among depressive fathers.

The impact of anxiety and depressive disorders in at least one parent before and after pregnancy for the physical and mental well-being of the family [e.g., (78, 79)] and the child [e.g., (80)] has been reported in a number of other studies. In our analysis, psychopathological load in couples was unrelated to partnership quality but related to unfavorable father-to-child attachment both ante- and postnatally. The activation and development of the caregiver abilities during the peripartum period in terms of feelings, cognitions, and infant related behaviors (i.e., parent-to-child attachment) is pivotal for later interaction with the child (81–83) and its development (84). Our findings add to conclusions from mother samples, that also in fathers, the development of antenatal attachment may be hampered by psychopathology and thereby affecting the child's development for fathers (85).

Along with findings from the MARI mother sample, results may indicate that lower partnership quality, lack of intimacy, and loss social support during peripartum increase perceived stress and pose mothers at risk for (post-partum) depressive disorders, unfavorable mother-child-interactions or regulation problems (regulatory disorders) in the child. A vicious circle may enfold, involving fathers to compensate. In case paternal support isn't enough, fathers are themselves at risk for mental disorders such as depressive or anxiety disorders, diminishing partnership quality and attachment to the child.

## Limitations

Interpretation of the observed associations should be made in respect to the strengths and weaknesses of the study's design, sampling, assessment, and analyzing procedures. This is one of the few prospective investigations on paternal perinatal mental health including a multiwave assessment to map the variety and multitude of experiences for expectant fathers. Retention rates of the study were generally high: 82.84% of fathers could be retained in the study until 4 months postpartum. However, selective drop out was observed for fathers with lower partnership quality at F-T1, likely leading to an underestimation of peripartum changes in partnership quality and its associations with father-to-child attachment. Rates for paternal depressive disorders prior to pregnancy were lower than lifetime rates in the general male population (86), while for anxiety disorders these rates were comparable. However, small numbers may have limited the fine-graded analyses. Further, other paternal disorders such as alcohol use disorder or externalizing behaviors were not taken into account. To ensure comparability to other studies, assessments were based on standardized and established diagnostic instruments. Although the German versions of both questionnaires for the assessment of father-to-child attachment were translated specifically for this study and replication and validation of findings in other (German) samples is warranted, the questionnaires have already demonstrated satisfactory to excellent psychometric properties and allow for a multifaceted evaluation of partnership quality, both cross-sectionally and prospectively. Sampling was restricted by inclusion criteria of the MARI main study, and the fact that fathers were only recruited given informed consent of the mother. Although unlikely, the incentive offered to recruit mothers and fathers might have introduced an additional bias. Despite its elaborate recruitment strategies, the MARI study was confronted with the typical problem of a higher representation of subjects with a good socio-economical background (i.e., higher educational levels, normal birth weight, higher social support, etc.) relative to the average population. This phenomenon showed even more in our subsample of parents who participated jointly. The sample of fathers may have differed from the general population in terms of time for study participation, work load, and single income pressure. For example, mothers with lower levels of stress or psychopathological strain were more likely to provide informed consent to contact their partner. Similarly, fathers with lower levels of strain were more likely to be interested in participating. Furthermore, parents who participated together were more often first-time parents and better educated. They also reported higher levels of social support and partnership quality. Thus, this subsample seemed to have the best conditions for a healthy transition from pregnancy to early infancy. Consequently, our results cannot inform about the relationships in high-risk families but rather shed some light on the relationships in a favorable environment and which variables should be addressed with regard to prevention.

## Conclusion

Transition into parenthood involves physiological, psychological, and social adjustments for the couple (87). Distressed parents may benefit from interdisciplinary support focusing on perinatal mental health and antenatal bonding, for example through interventions that aim to strengthen the couple's relationship, provide strategies to cope with postpartum sleep deprivation, promote parental task sharing after birth, or encourage supportive communication skills in partners to relieve feelings of parental unworthiness or anger toward the child. Still, more research is warranted to determine how couples at risk can be attracted to actually participate in clinical psychological research and interventions to understand and improve their emerging relationships during the transition to parenthood and thus promote (mental) health development in the families.

## Data Availability Statement

The datasets presented in this article are not readily available because of legal and ethical constraints. Public sharing of participant data was not included in the informed consent of the study. Requests to access the datasets should be directed to Julia Martini, julia.martini@uniklinikum-dresden.de.

## Ethics Statement

The studies involving human participants were reviewed and approved by Ethics Committee of the Medical Faculty of the Technische Universität Dresden (No: EK 94042007). The patients/participants provided their written informed consent to participate in this study.

## Author Contributions

SK, HC-P, and IH: prepared the manuscript. SK, HC-P, IH, JP, JW, and JM: analyses and interpretation of the data. JP, JM, and SG-N: helped to draft the manuscript. JP, JW JM, and SG-N: critically revised the manuscript for important intellectual content. All authors contributed to the article and approved the submitted version.

## Conflict of Interest

The authors declare that the research was conducted in the absence of any commercial or financial relationships that could be construed as a potential conflict of interest.
