# Corrigendum: Associations of Partnership Quality and Father-to-Child Attachment During the Peripartum Period. A Prospective-Longitudinal Study in Expectant Fathers

**DOI:** 10.3389/fpsyt.2021.790327

**Published:** 2022-01-11

**Authors:** Susanne Knappe, Johanna Petzoldt, Susan Garthus-Niegel, Julia Wittich, Hans-Christian Puls, Isabell Huttarsch, Julia Martini

**Affiliations:** ^1^Institute of Clinical Psychology and Psychotherapy, Technische Universität Dresden, Dresden, Germany; ^2^Department of Medicine, Faculty of Medicine, Medical School Hamburg, Hamburg, Germany; ^3^Department of Child Health and Development, Norwegian Institute of Public Health, Oslo, Norway; ^4^Institute and Policlinic of Occupational and Social Medicine, Faculty of Medicine, Technische Universität Dresden, Dresden, Germany; ^5^Department of Psychiatry and Psychotherapy, Faculty of Medicine, Carl Gustav Carus University Hospital, Technische Universität Dresden, Dresden, Germany

**Keywords:** paternal attachment, paternal anxiety or depression, partnership quality, peripartum, fatherhood, pregnancy, postpartum

In the original article, there was a mistake in Figure 1 as published. The figure presented refers to a different publication (10.3389/fpsyt.2020.554221). The corrected Figure 1 appears below.

**Figure 1:**
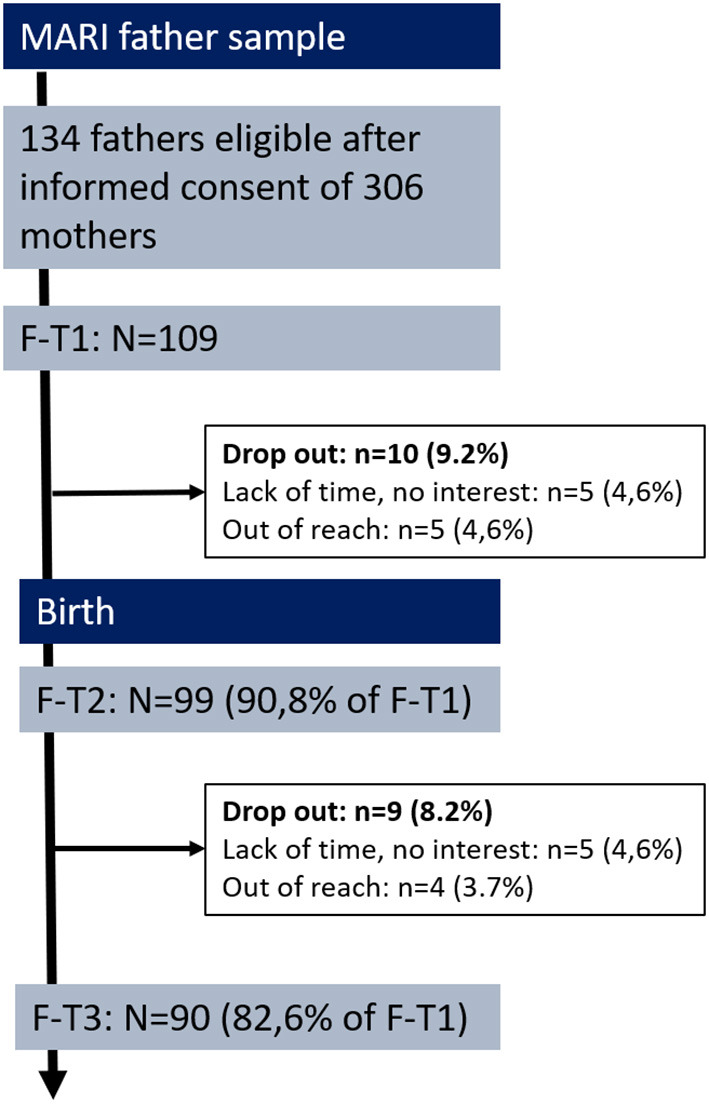
Flow chart and retension rates of the MARI father study. F-T1 week 22 to 24 of gestation, F-T2 at 10 days postpartum, F-T3 at 4 months postpartum.

The authors apologize for this error and state that this does not change the scientific conclusions of the article in any way. The original article has been updated.

## Publisher's Note

All claims expressed in this article are solely those of the authors and do not necessarily represent those of their affiliated organizations, or those of the publisher, the editors and the reviewers. Any product that may be evaluated in this article, or claim that may be made by its manufacturer, is not guaranteed or endorsed by the publisher.

