# Supplementary material for: Associations of Partnership Quality and Father-to-Child Attachment During the Peripartum Period. A Prospective-Longitudinal Study in Expectant Fathers
Source: Front Psychiatry. 2021 Apr 20;12:572755. doi: 10.3389/fpsyt.2021.572755 (PMC8093807; doi:10.3389/fpsyt.2021.572755)
Supplement: Supplementary file 1 [file Data_Sheet_1.pdf]

Supplemental material.

Supplemental Table S1. Sociodemographic description of mothers, fathers and infants (*n*=306)

	Total sample (*n* = 306)		Mother sample (*n* = 197)		Parents sample (*n* = 109)		Test characteristics	
							parameter	p-value
Maternal age in years (*n*=306)	mean=28.0	SD=4.4	mean=28.2	SD=4.2	mean=27.7	SD=4.6	t=1.02	<0.05
Paternal age in years (*n*=109)	-	-	-	-	mean=31.1	SD=5.8	-	-
Maternal education (*n*=306)								
≤10y of education	n=98	32.0%	n=71	36.0%	n=27	24.7%	chi2=4.09	<0.05
>10y of education	n=208	68.0%	n=126	64.0%	n=82	75.2%		
Paternal education (*n*=306)								
≤10y of education	n=125	40.8%	n=92	46.7%	n=33	30.3%	chi2=7.84	<0.01
>10y of education	n=181	59.2%	n=105	53.3%	n=76	69.7%		
Maternal status of employment (*n*=306)								
not employed	n=30	9.8%	n=24	12.2%	n=6	5.5%	chi2=3.54	n.s.
employed	n=276	90.2%	n=173	87.8%	n=103	94.5%		
Paternal status of employment (*n*=306)								
not employed	n=15	4.9%	n=9	4.6%	n=6	5.5%	chi2=0.13	n.s.
employed	n=291	95.1%	n=188	95.4%	n=103	94.5%		
Marital status(*n*=306)								
not married	n=193	63.1%	n=122	61.9%	n=71	65.1%	chi2=0.31	n.s.
married	n=113	36.9%	n=75	38.1%	n=38	34.9%		
Parity(*n*=306)								
primiparous mother	n=178	58.2%	n=104	52.8%	n=74	67.9%	chi2=6.57	<0.01
multiparous mother	n=128	41.8%	n=93	47.2%	n=35	32.1%		
Number of sibblings (*n*=279)								
0	n=151	54.1%	n=84	48.3%	n=67	36.2%	chi2=6.36	<0.05
1 or more	n=128	45.9%	n=90	51.7%	n=38	63.8%		
Prematurity (*n*=286)								
>37 weeks gestational age	n=11	3.9%	n=9	5.0%	n=2	1.9%	chi2=1.81	n.s.
≤37 weeks gestational age	n=175	96.1%	n=170	95.0%	n=105	98.1%		
Mode of delivery (*n*=286)								
spontaneous vaginal delivery	n=225	78.7%	n=137	76.5%	n=88	82.2%	chi2=1.30	n.s.
assisted delivery/ c-section	n=61	21.3%	n=42	23.5%	n=19	17.8%		

Infant sex (*n*=286)								
boys	n=147	51.4%	n=86	48.0%	n=61	57.0%	chi2=2.15	>0.1
girls	n=139	48.6%	n=93	52.0%	n=46	43.0%		
**Infant birth weight in kg (*n*=286)**	mean=3.44	SD=0.5	mean=3.36	SD=0.5	mean=3.58	SD=0.4	**t=-4.05**	**<0.001**
Breastfeeding (*n*=286)								
exclusive at 4m pp	n=219	76.6%	n=132	73.7%	n=87	81.3%	chi2=2.14	n.s.
no or non-exclusive at 4m pp	n=67	23.4%	n=47	26.3%	n=20	18.7%		
Maternal social support at 22-24w GA (*n*=293)	mean=4.4	SD=0.5	mean=4.3	SD=0.5	mean=4.4	SD=0.5	t=-1.79	n.s.
Paternal social support at 22-26w GA (*n*=103)	-	-	-	-	mean=4.3	SD=0.6		
**Maternal social support at 4m pp (*n*=281)**	mean=4.3	SD=0.6	mean=4.3	SD=0.7	mean=4.4	SD=0.5	**t=-1.98**	**<0.05**
Paternal social support at 4m pp (*n*=80)	-	-	-	-	mean=4.2	SD=0.5		
**Maternal partnership quality at 22-24w GA (*n*=285)**	mean=70.6	SD=12.3	mean=69.1	SD=12.9	mean=73.1	SD=10.8	**t=-2.74**	**<0.01**
Paternal partnership quality at 22-26w GA (*n*=103)	-	-	-	-	mean=67.2	SD=10.9		
**Maternal partnership quality at 4m pp (*n*=272)**	mean=66.7	SD=13.6	mean=64.9	SD=14.5	mean=69.6	SD=11.5	**t=-2.75**	**<0.01**
Paternal partnership quality at 4m pp (*n*=79)	-	-	-	-	mean=66.8	SD=12.1		

m months, n number, n.s. not significant, pp postpartum SD standard deviation, t T-value, y years, bold prints indicate statistical significance at p < .05

Supplemental material

Supplemental Table S2. Means and standard deviations for partnership quality at F-T1, F-T3 and across time

	**Fathers at F-T1 (antenatal)**				**Fathers at F-T3 (postnatal)**				**difference F-T3 minus F-t1**		
Partnership quality (PFB)	Mean	SD	min	max	Mean	SD	min	max	t	df	p
Sum score	68,12	10,6	39	89	66,54	12,24	29	90	2	75	0,083
Communication	22,78	3,88	12	30	22,63	4,56	9,00	30	0,38	75	0,708
Quarreling	5,17	3,92	0	15	5,79	4,59	0,00	18	-1,64	75	0,105
Tenderness	20,51	5,31	3	29	19,7	6,00	4,00	30	1,88	75	0,063
Satisfaction	4,453	0,66	2	5	4,22	0,83	1,00	5	2,87	73	**0,005**

PFB partnership quality
SD standard deviation, min minimum sore, max maximum score, t T-Test, df degrees of freedom, p significance level (one-tailed) at p<.01

Supplemental material

Supplemental Table S3. Prospective associations between psychopathological load and partnership characteristics

		Prospective associations with F-T3					Prospective associations with changes across peripartum (F-T3 - F-T1)				
		b	t	p	95%CI		b	t	p	95%CI	
PFB sum score[1]	one parent affected	0,12	-0,44	0,663	-0,68	0,43	0,17	0,67	0,506	-0,34	0,69
	both parents affected	0,30	-0,94	0,351	-0,92	0,33	0,18	0,63	0,531	-0,4	0,76
Communication	one parent affected	0,00	0,00	0,997	-2,19	2,18	0,11	0,15	0,878	-1,32	1,54
	both parents affected	-1,07	-0,87	0,389	-3,52	1,39	-0,13	-0,17	0,868	-1,74	1,48
Quarreling[1]	one parent affected	-0,10	-0,45	0,655	-0,53	0,345	-0,05	-0,24	0,813	-0,45	0,35
	both parents affected	-0,07	-0,28	0,778	-0,56	0,42	0,61	0,27	0,786	-0,39	0,51
Tenderness[1]	one parent affected	-0,24	-0,82	0,416	-0,82	0,34	0,21	0,78	0,437	-0,32	0,73
	both parents affected	-0,40	-1,20	0,221	-1,06	0,25	0,42	0,14	0,159	-0,17	1,10
Satisfaction	one parent affected	-0,17	-0,72	0,475	-0,65	0,31	-0,13	-0,65	0,521	-0,55	0,28
	both parents affected	-0,16	-0,59	0,559	-0,69	0,38	0,01	0,05	0,963	-0,45	0,47

[1] results based on robust regression.

PFB partnership quality questionnaire

pure A anxiety disorder only, pure D depressive disorder only, comorbid AD comorbid anxiety and depressive disorder, F-T1 week 22 to 24 of gestation, F-T3 at 4 months postpartum

b unstandardised regression coefficient, CI confidence interval, p significance level at 0.05, bold prints indicate statistical significance at $p < .05$

Supplemental material

Supplemental Table S4. Means and standard deviations for father-to-infant attachment at F-T1 and F-T3

Father-to-infant attachment	Mean	SD	min	max
antenatal attachment (PAAS at F-T1)				
sum score	62,34	5,78	48	75
quality of attachment	35,38	2,79	28	40
intensity of attachment	18,36	3,11	12	27
postnatal attachment (PPAS at F-T3)				
sum score	75,70	8,72	52	92
patience and tolerance	32,17	4,13	24	40
pleasure in interaction	24,72	4,27	13	32
affection and pride	18,81	1,68	12	20

PAAS antenatal father-to-child-attachment, PPAQ postnatal father-to-child attachment, F-T1 week 22 to 24 of gestation, F-T3 at 4 months postpartum, SD standard deviation, min minimum sore, max maximum score

Supplemental material

Supplemental Table S5. Prospective associations between psychopathological load and father-to-child attachment

		N	mean	SD	b	t	p	95%CI	
**Antenatal attachment (PAAS)**									
PAAS sum score	no parent affected	19	63,74	5,72					
	one parent affected	37	6,26	5,20	-1,17	-0,72	0,473	-4,40	2,06
	both parents affected	20	60,60	6,65	-3,14	-1,71	0,092	-6,80	0,53
PAAS quality of attachment	no parent affected	19	36,16	2,75					
	one parent affected	37	35,57	2,46	-0,59	-0.76	0,447	-2,13	0,95
	both parents affected	20	34,30	3,18	**-1,86**	**-2.12**	**0,037**	**-3,60**	**-0,11**
PAAS intensity of attachment	no parent affected	19	19,00	3,33					
	one parent affected	37	18,27	2,77	-0,73	-0,83	0,410	-2,49	1,03
	both parents affected	20	17,90	3,52					
**Postnatal attachment (PPAS)**									
PPAS sum score	no parent affected	19	77,36	8,76					
	one parent affected	37	76,61	7,83	-0,76	-0,31	0,757	-5,60	4,09
	both parents affected	20	72,46	9,79	-4,91	-1,78	0,079	-1,04	0,59
PPAS patience and tolerance	no parent affected	19	31,95	4,27					
	one parent affected	37	32,82	4,14	0,87	0,75	0,457	-1,45	3,19
	both parents affected	20	31,16	3,93	-0,79	-0,60	0,550	-3,42	1,84
PPAS pleasure in interaction	no parent affected	19	26,38	4,26					
	one parent affected	37	24,79	3,87	-1,59	-1,36	0,178	-3,93	0,74
	both parents affected	20	23,03	4,52	**-3,35**	**-2,52**	**0,014**	**-6,00**	**-0,70**
PPAS affection an pride	no parent affected	19	19,03	1,28					
	one parent affected	37	19,00	1,38	-0,03	-0,07	0,947	-0,97	0,91
	both parents affected	20	18,27	2,35	-0,77	-1,43	0,156	-1,83	0,30

PAAS antenatal father-to-child-attachment, PPAQ postnatal father-to-child attachment, pure A anxiety disorder only, pure D depressive disorder only, comorbid AD comorbid anxiety and depressive disorder

b unstandardised regression coefficient, CI confidence interval, M mean, SD Standard deviation, t T-Test, p significance level at 0.05, bold prints indicate statistical significance at $p < .05$
